# Origin of the domestic chicken from modern biological and zooarchaeological approaches

**DOI:** 10.1093/af/vfab016

**Published:** 2021-06-19

**Authors:** Masaki Eda

**Affiliations:** Hokkaido University Museum, Hokkaido University, Kita 10, Nishi 8, Kita-ku, Sapporo Hokkaido 060-0810, Japan

**Keywords:** chicken, DNA analysis, domestication, medullary bone, red junglefowl, zooarchaeology

**Implications**

- Chickens (*Gallus gallus domesticus* or *Gallus domesticus*) are the most common domestic animals worldwide. However, the origin of their domestication is obscure.
- The early 21st century mitochondrial DNA data suggest that various red junglefowl (*Gallus gallus*) subspecies are the wild ancestors of the domestic chicken. However, more recent genomic data reveals that *Gallus gallus spadiceus*, indigenous to northern Thailand, Myanmar, and southwestern China, is its main wild ancestor.
- Domestic chicken bones are detected at early and middle Holocene archaeological sites. However, their authenticity remains controversial, with direct radiocarbon dating and reliable species identification being required.
- The first red junglefowl domestication might have occurred within the native range of the species, especially in the distribution area of *G. g. spadiceus*. Because archaeological red junglefowl may have been present during the various domestication stages, it is believed that its bone analyses will clarify their historical role and relationship with humans in the region.

## Introduction

Chickens (*Gallus gallus domesticus* or *Gallus domesticus*) are the most common domestic animals worldwide. In 2017, the global chicken population was >22 billion (FAO, 2020; http://www.fao.org/poultry-production-products/production/poultry-species/chickens/en/). They are bred on all continents and countries except Antarctica and Vatican City (Lawler, 2015). To meet the growing demand for animal foods, high-yielding commercial chicken breeds were developed in recent decades for meat and egg production. Nearly 1,600 different local chicken breeds are internationally recognized (FAO, 2020).

Despite their global distribution, the origin of chicken domestication remains obscure. Two approaches have been used to investigate this subject. First, their morphological, ecological, and genetic characteristics were compared with those of other species using modern biological techniques. Second, the characteristics of the chickens were reconstructed for each era and region using zooarchaeological remains. Herein, prior research on the origin of global chicken domestication using modern biological and zooarchaeological approaches were reviewed, and future perspectives for studies on the origin of domestic chicken were discussed.

## Modern Biological Approach: What is the Wild Ancestor of Domestic Chicken?

### Single-species vs. multispecies origin of domestic chicken

**Figure 1.**
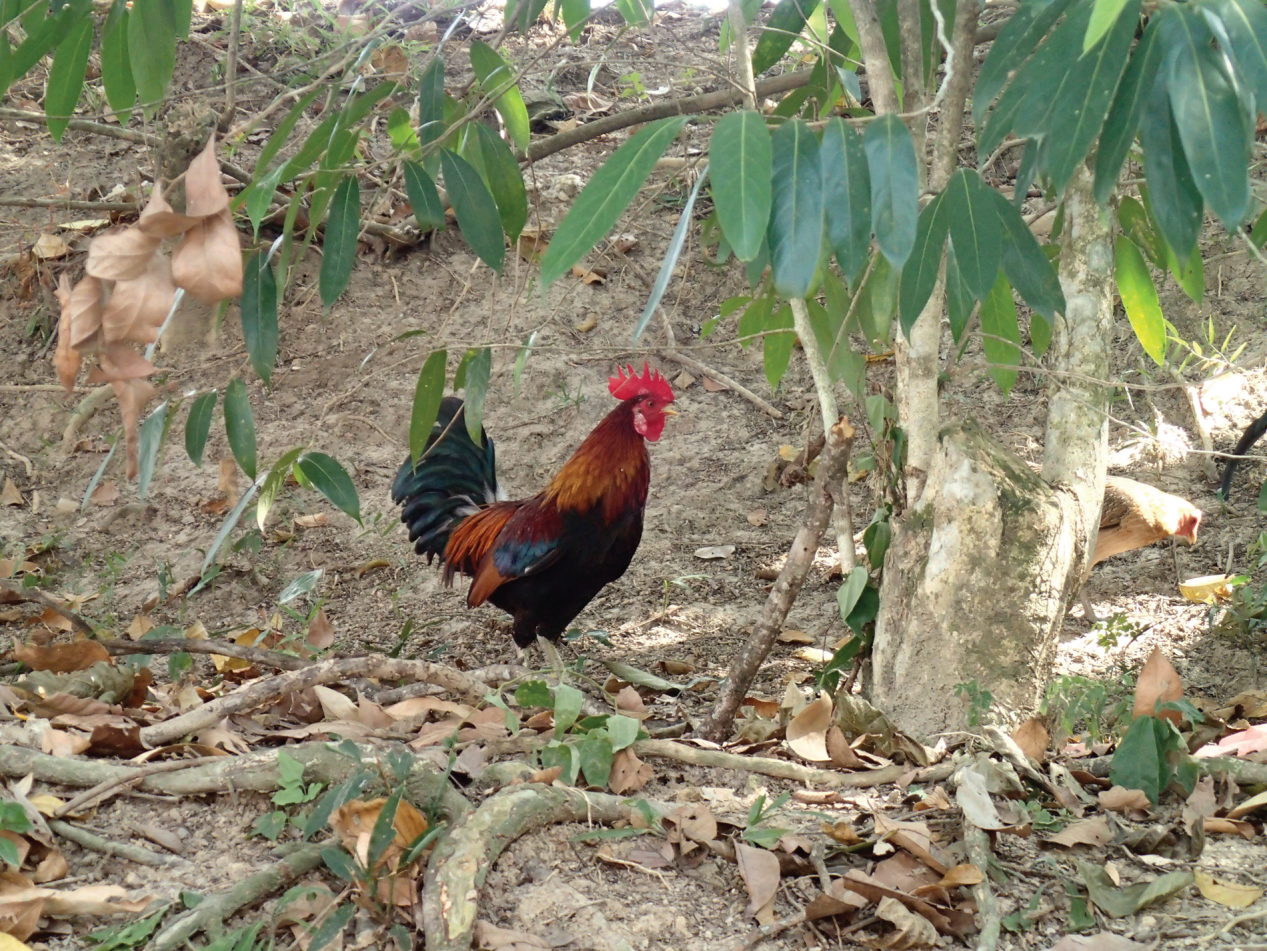
Wild red junglefowl in Kuala Lumpur, Malaysia. Photograph taken by the author.

Charles Darwin proposed that *Gallus bankiva* (current *Gallus gallus*, red junglefowl; Figure 1) was the ancestor of domestic chickens based on several lines of evidence: 1) the extremely close resemblance between red junglefowl and the game fowl (the most typical domestic fowl) regarding color, general structure, and voice; 2) their fertility, when the red junglefowl and game fowl were crossed; 3) the possibility of the wild red junglefowl being tamed; and 4) the broad phenotypic variation of the wild red junglefowl (Darwin, 1868). Moreover, Darwin rejected the possibility that the other three *Gallus* wild junglefowl (Ceylon junglefowl (*Gallus lafayetii*), gray junglefowl (*Gallus sonneratii*), and green junglefowl (*Gallus varius*)) could be the primitive stocks of the domestic chicken as hybrids derived from these species crossed with the domestic chicken were usually infertile.

Darwin assertions about the origins of domestic chickens were widely accepted and certain researchers considered the matter settled (e.g., Beebe, 1921). Nevertheless, others suggested a polyphyletic origin of domestic chicken, including two possibilities: 1) all are descendants of two or more of the four extant wild junglefowl species or 2) Mediterranean breeds, such as white leghorns, may in fact be red junglefowl descendants, whereas Asiatic breeds, such as Cochins, Brahmas, and Langshans, may have originated from some other extinct ancestors (Hutt, 1949). The second scenario could be explained by the difference between existing junglefowl (as well as Mediterranean breeds) and Asiatic breeds regarding their morphophysiological traits and temperament. Apart from the possibilities that certain extinct ancestors may have given rise to Asiatic breeds, the polyphyletic origin of domestic chicken was indicated by phenotypic characteristics that could have derived from other wild junglefowl (e.g., extended black plumage may have originated from green junglefowl and yellow skin may have originated from gray junglefowl) and given the fact that hybrids of any *Gallus* wild junglefowl and domestic chicken were in some cases fertile (Hutt, 1949).

### Evidences of single-species origin of domestic chicken

Molecular analyses revealed a close genetic relationship between domestic chicken and red junglefowl, which harbor very similar egg proteins (Baker, 1968). In contrast, their G_2_ globulin was distinct from that of gray junglefowl. Hence, red junglefowl might be the main progenitor of domestic chicken (Baker, 1968). The close relatedness between the domestic chicken and red junglefowl was further demonstrated by phylogenetic analyses of the domestic chicken and four *Gallus* junglefowl via blood protein and DNA fingerprinting. Moreover, analyses of the 400 base-pair (bp) nucleotide sequence of the mitochondrial DNA (mtDNA) control region in four wild junglefowl species and nine domestic chicken breeds revealed a monophyletic relationship between domestic chicken and red junglefowl (Fumihito et al., 1996).

### Single-subspecies versus multisubspecies origin of domestic chicken

**Figure 2.**
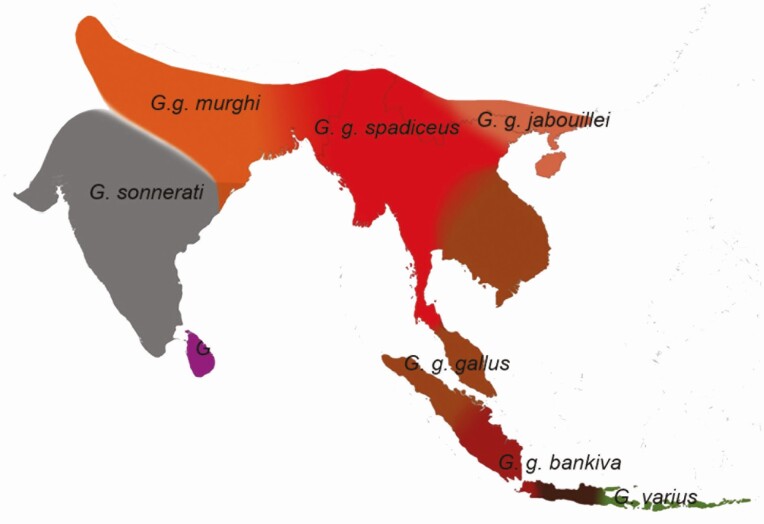
Map of the geographic distribution of the wild junglefowl according to Wang et al. (2020).

There are five extant red junglefowl subspecies: *Gallus gallus gallus*, *Gallus gallus spadiceus*, *Gallus gallus jabouillei*, *Gallus gallus murghi*, and *Gallus gallus bankiva* (Figure 2). However, there are morphological intergradations among the four continental subspecies. Fumihito et al. (1996) reported that *G. g. bankiva* was distinct from *G. g. spadiceus* and *G. g. gallus*. They also showed that the nine domestic chicken breeds and the continental *G. g. gallus* population in Southeast Asia formed a single cluster in the phylogenetic tree. Hence, that population might be the sole ancestor of all domestic chicken breeds, originating from a single domestication event in Thailand and adjacent regions (Fumihito et al., 1996).

**Figure 3.**
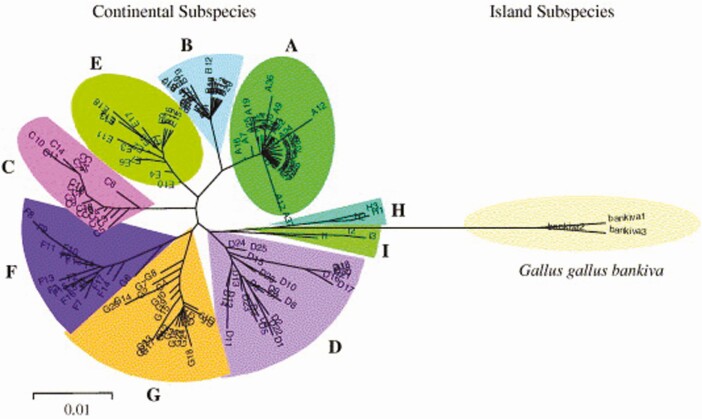
Unrooted neighbor-joining tree of 169 haplotypes from 834 domestic chickens and 66 red junglefowl obtained by Liu et al. (2006).

Nonetheless, Liu et al. (2006) indicated that Fumihito et al. (1996) lacked the analysis of domestic chicken breeds and wild red junglefowl subspecies from China and India and comprised small sample sizes. To overcome these constraints, Liu et al. (2006) analyzed the partial mtDNA control regions of 834 domestic chickens across Eurasia and of 66 red junglefowl, including four subspecies but not *G. g. murghi*. The phylogenetic analysis revealed two main clades of which were formed by *G. g. bankiva* sequences and by sequences of other continental subspecies and domestic chickens (Liu et al., 2006) (Figure 3). The latter consisted of nine highly divergent mtDNA clades (A–I). *Gallus g. spadiceus* and *G. g. jabouillei* were observed mainly in clades A, B, and F, whereas *G. g. gallus* was observed mainly in clades D, H, and I. Clades A–G and I included domestic chickens. Clades A, B, and E were ubiquitously distributed among Eurasian chickens, whereas the others were mainly confined to South and Southeast Asian chickens. Clades F and G were mostly restricted to Yunnan, whereas clade C was distributed over southern and southeastern China and Japan. Based on these distinct distribution patterns and population expansion signature of each clade, Liu et al. (2006) suggested that various clades may have originated from different regions and multiple independent domestication events might have occurred. The multiple domestication event hypothesis was supported by additional sampling of *G. g. murghi* and domestic chickens from India, with extensive mtDNA control region analysis of 4,732 domestic chickens and 206 red junglefowl and 61 mtDNA genome studies of representative haplotypes (Miao et al., 2013).

### Evidences of multispecies origin of domestic chicken

Nonetheless, genetic analysis of the yellow skin pigmentation, which is common to numerous commercial chicken breeds, revealed that the red junglefowl was not the sole wild ancestor of the domestic chicken (Eriksson et al., 2008). Domestic chickens with yellow skin are homozygous for a recessive allele, whereas white-skinned chickens bear one or more dominant allele. Eriksson et al. (2008) showed that the recessive allele associated with yellow skin was caused by regulatory mutation(s) in the dermal β-carotene dioxygenase 2 (*BCDO2*). Phylogenetic analysis of chickens and four wild junglefowl based on a 23.8-kb sequence comprising the *BCDO2* locus showed that white-skinned breeds clustered with red junglefowl, whereas yellow-skinned breeds clustered with gray and green junglefowl. Thus, the yellow skin allele might have originated from a different species, most likely gray junglefowl (Eriksson et al., 2008).

The polyphyletic species origin of domestic chicken was corroborated by recent genome-wide studies. Lawal et al. (2020) analyzed the genomes of 53 indigenous village chickens, nine red junglefowl, as well as three other junglefowl species and the common pheasant (Phasianus colchicus). The data suggested that the red junglefowl was the main ancestral species of domestic chickens and their divergence of domestic chickens and red junglefowl may have occurred 8,093 years ago (range: 7,014–8,768 years). There was also extensive bidirectional introgression between the gray junglefowl and domestic chickens, a few introgression signatures between domestic chickens and Ceylon junglefowl, and a single introgression signature between domestic chickens and green junglefowl (Lawal et al., 2020).

**Figure 4.**
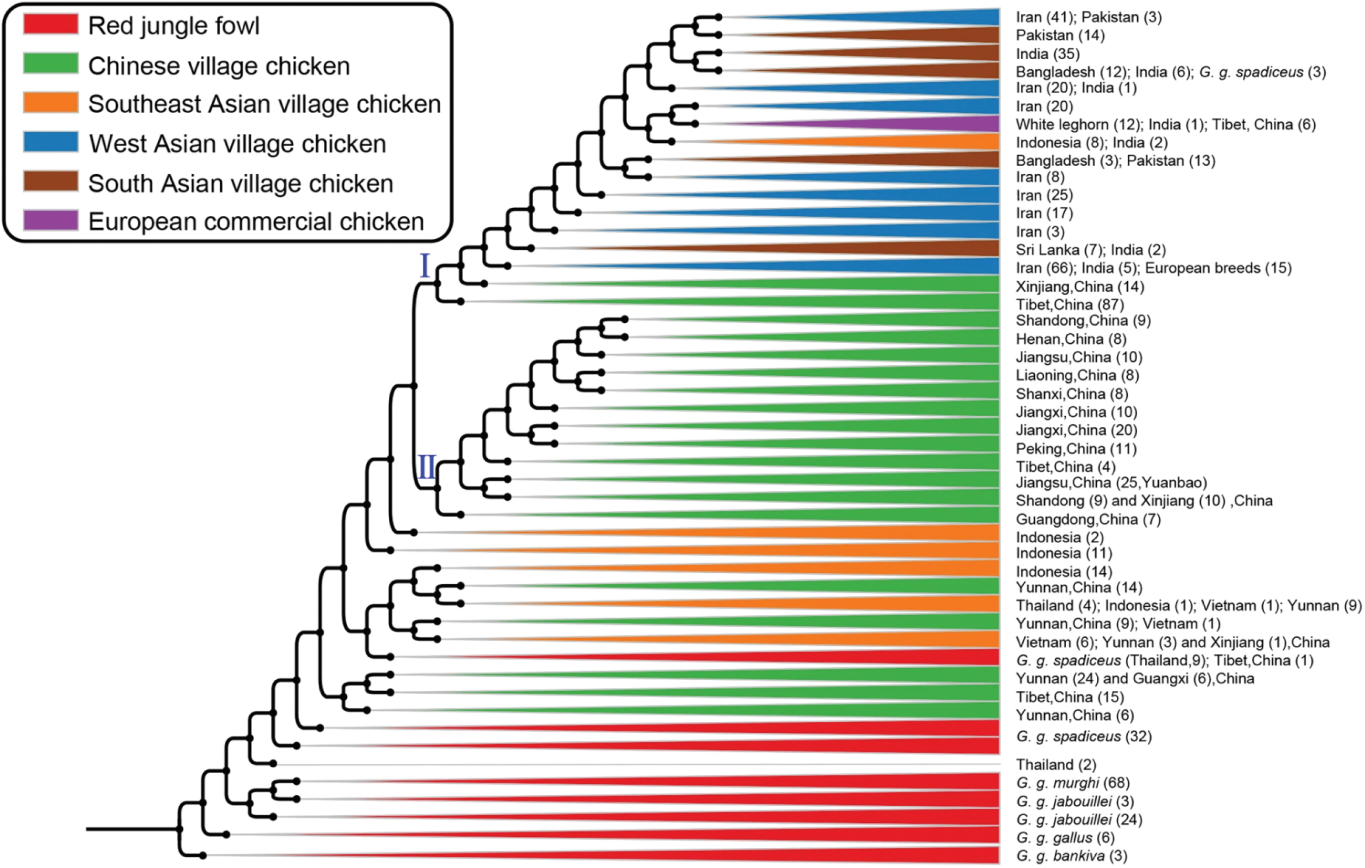
Maximum-likelihood phylogenetic tree showing the monophyletic clade formed by domestic chickens with *Gallus gallus spadiceus* as the nearest wild progenitor. Black dots at nodes indicate ≥99% bootstrap support. Domestic chicken and red junglefowl clades are collapsed and colored according to geographic range and subspecies classification (Wang et al., 2020).

Wang et al. (2020) analyzed 863 genomes from worldwide sampling of chickens, representatives of all four *Gallus* junglefowl species and of all five red junglefowl subspecies. In the phylogenetic tree, all domestic chickens formed a monophyletic clade with *G. g. spadiceus* (Figure 4). In addition, a principal component analysis disclosed relatively closer genetic affinity between the domestic chicken and *G. g. spadiceus*, suggesting that the subspecies was their closest progenitor. A molecular clock analysis further indicated that the domestic chicken diverged from *G. g. spadiceus* 9,500 ± 3,300 years ago, although this point does not necessarily correlate with the beginning of the domestication process. Once again, there was evidence of admixture between other junglefowl species and domestic chickens. However, the introgression fragments occurred at a very low frequency and were confined mainly to local chickens inhabiting the native ranges of all local wild junglefowl, except gray junglefowl (Wang et al., 2020). The authors concluded that the domestic chickens were initially derived from *G. g. spadiceus* in southwestern China, northern Thailand, and Myanmar, translocated across Southeast and South Asia, and interbred with other local red junglefowl subspecies and junglefowl species (Wang et al., 2020). Wang et al. (2020) also indicated that previous studies using mtDNA analysis were unable to confirm the origins of domestic chickens owing to recurrent hybridizations shared mtDNA from wild relatives and domestic chickens.

### Summary

Based on its morphological, physiological, and behavioral traits, red junglefowl was considered the main ancestor of domestic chicken in the late 19th century (Darwin, 1868), which was supported by molecular data collected in the 20th century (Baker, 1968; Fumihito et al., 1996). However, the mtDNA analyses from the early 21st century suggested multiple *G. gallus* subspecies as the wild ancestors of the domestic chicken (Liu et al., 2006; Miao et al., 2013). Moreover, recent genome data revealed that the red junglefowl subspecies *G. g. spadiceus* is the main wild ancestor, which was translocated across Southeast and South Asia and locally interbred with other red junglefowl subspecies and junglefowl species (Wang et al., 2020). This hypothesis explains the origin of certain morphological features, such as yellow skin, derived from the gray junglefowl and currently present in domestic chickens but absent in red junglefowls (Eriksson et al., 2008).

## Zooarchaeological Approach: Where can the Oldest Domestic Chicken Bones be Found?

### Candidate sites for the oldest domestic chicken bone

**Figure 5.**
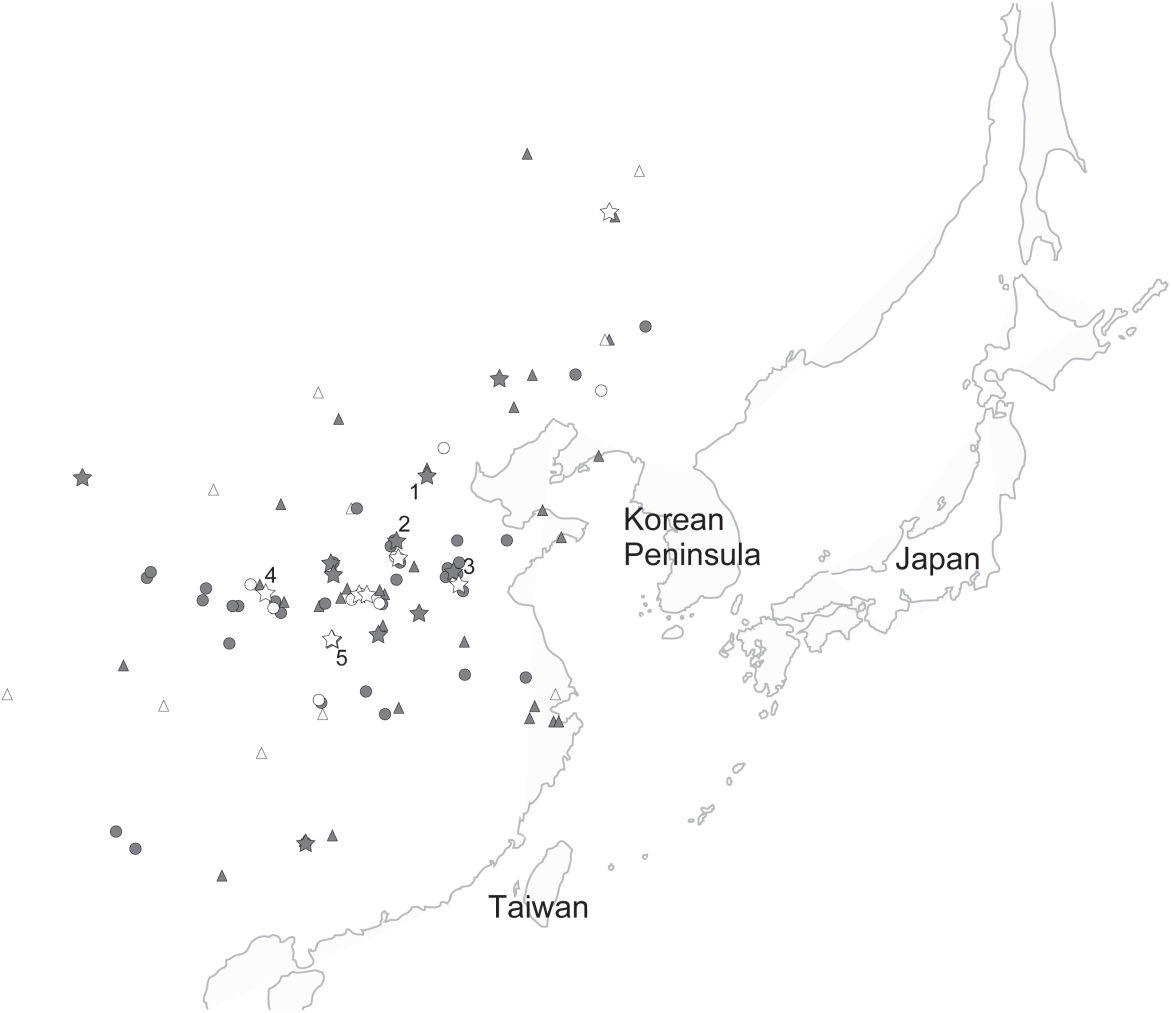
Previously reported archaeological sites of chicken bones. Sites with (circles) and without (triangles) chicken bones from the Neolithic (gray) and Bronze Age (white) in China (after Eda et al., 2016) are indicated. Sites reported by Eda et al. (2016) (star; 1. Nanzhuangtou; 2. Cishan; 3. Wangyin; 4. Zaoshugounao; 5. Xiawanggang) are also shown. Of note, candidate chicken bones were found only in Zaoshugounao and Xiawanggang.

Frederick Zeuner suggested that chickens were first domesticated in the Indus Valley region ca. 2000 BC (Zeuner, 1963) based on seals and figurines depicting chicken and a chicken femur found at Mohenjo-Daro, Pakistan (Sewell and Guha, 1931). In 1988, West and Zhou (1988) reviewed archaeological sites with chicken bones from before the 1st century AD. They listed 90 archaeological sites containing chicken bones in Europe, the Middle East, South Asia, and East Asia. They introduced Cishan (Hebei Province; 5405 ± 100 to 5285 ± 105 BC), Peiligang (Henan Province; 5935 ± 480 to 5495 ± 200 BC) (Figure 5), and 16 other Neolithic Chinese sites predating Mohenjo-Daro and concluded that chickens were first domesticated in Southeast Asia, transported north, and established in China (West and Zhou, 1988).

Phasianidae bones from Cishan were identified as domesticated chickens for three reasons (Zhou, 1981). First, Cishan is located far north of the natural distribution range of wild red junglefowl, which is restricted to southern China. Second, tarsometatarsi with spurs were more abundant at that site than tarsometatarsi without spurs. As, in general, male birds have spurs but females do not, Zhou (1981) discussed a possible male-biased chicken butchering and regarded it as proof of domestication. Third, spurred tarsometatarsi from Cishan (range: 72.0–86.5 mm; mean: 79.0 mm) were, on average, longer than those of modern male wild red junglefowl (range: 70.0–82.0 mm; mean: 78.7 mm). According to West and Zhou (1988), northern China can be one of the early centers of chicken domestication (with some caution) given that the numerous putative chicken bones have been recorded at several archaeological sites (Serjeantson, 2009).

Xiang et al. (2014) published an intriguing article on early Holocene domestic chicken in northern China. Phasianidae bones from Nanzhuangtou (*n* = 22; Hebei Province; 10000–7600 BC), Cishan (*n* = 7), Wangyin (*n* = 6; Shandong Province; 4500–3500 BC), and Jiuliandun (*n* = 4; Hebei Province; 500–200 BC) were subjected to ancient DNA analysis. Species identification using a 159-bp fragment of the mtDNA cytochrome c oxidase subunit I gene revealed that each of the 13 sequences obtained (seven from Nanzhuangtou, one from Cishan, three from Wangyin, and two from Jiuliandun) were identified as *Gallus* since they were closer to *Gallus* than to any other genus, such as *Phasianus*, *Alectoris*, *Lophura*, *Tetraophasis*, and *Syrmaticus*.

Xiang et al. (2014) also succeeded in the analysis of a 326-bp fragment of the mtDNA control region in eight samples (three from Nanzhuangtou, one from Cishan, two from Wangyin, and two from Jiuliandun). The median-joining network constructed based on the obtained sequences, 10 published ancient chicken sequences, and 1,001 extant published sequences from four *Gallus* species showed that all samples were included in three of the five main haplogroups of modern domestic chickens. Thus, the bones originated from domestic chicken. As there were abundant remains of tropical animal and plant species excavated at Cishan and Nanzhuangtou, Xiang et al. (2014) estimated that the North China Plain was warmer and more humid with much larger forest cover and was a suitable habitat for junglefowl during the early Holocene. They concluded that the distribution range of the wild red junglefowl was far wider in the early Holocene than in the present and that domestic chicken farming began ~10,000 years ago in northern China (Xiang et al., 2014).

### Challenges to the early and middle Holocene chicken domestication in northern China

The report by Xiang et al. on domestic chickens in northern China during the early and middle Holocene was questioned in two subsequent papers. Peng et al. (2015) criticized the improper incorporation of the primer in the analyzed DNA sequence and the insufficiency of the analyzed sequence length. Xiang et al. (2015b) admitted the mistake, but they insisted that the main conclusion was unchanged by these limitations. In turn, Peters et al. (2015) also raised several questions: improper incorporation of the primer sequence, unsuitability of the climatic condition of northern China for red junglefowl, difficulty of ancient DNA sequence amplification based on the thermal age calculation, suspicion on the morphological identification of the analyzed samples, and possibility of contamination from the later cultural deposits. Xiang et al. (2015a) contradicted these questions and concluded “further discussions confirm early Holocene chicken domestication in northern China” in the title of their reply letter.

Several studies challenged the hypothesis that early and middle Holocene chicken domestication and Neolithic poultry husbandry occurred in northern China. Pitt et al. (2016) estimated the suitability of the modern and mid-Holocene (ca. 4000 BC) of this region for red junglefowl distribution and found that the climate of northern China did not meet these requirements during either period. Furthermore, Huang et al. (2018) analyzed the modern domestic chicken mtDNA with a focus on certain haplogroups that were absent in red junglefowl samples and were restricted to domestic chickens. They disclosed a recent domestic chicken expansion in northern China from a maternal perspective and denied early Holocene chicken domestication in this region. Following their review of Holocene paleoclimate and archaeofaunal archives, Peters et al. (2016) concluded that the habitat requirements of (sub-)tropical red junglefowl were absent during the early and middle Holocene of northern China. They also suggested that the middle Yangtze River basin delimited the northernmost boundary of a thermally optimal habitat for red junglefowl during the Holocene.

Eda et al. (2016) analyzed 280 Phasianidae hindlimb bones (femur, tibiotarsus, and tarsometatarsus) from 11 Neolithic sites, including Nanzhuangtou (*n* = 7), Cishan (*n* = 5), and Wangyin (*n* = 70), and eight Bronze Age sites in China (Figure 5), based on the morphological identification criteria. Because their approach was focused on the discrimination of chickens and red junglefowl from indigenous Japanese pheasants, they were unable to distinguish chickens and red junglefowl from the other 62 indigenous Phasianidae species in China. Nevertheless, the criteria used were useful to exclude nonchicken and nonred junglefowl bones, identifying candidate chicken or red junglefowl bones (Eda et al., 2016). Only one potential chicken bone was identified from the Neolithic period (at Xiawanggang) and only two potential chicken bones were identified from the early Bronze Age (at Zaoshugounao). The other Phasianidae bones, including those from Nanzhuangtou, Cishan, and Wangyin, were identified as nonchicken bones. Therefore, these findings suggest that chickens were not widely kept and red junglefowl were not extensively distributed throughout central and northern China during the early and middle Holocene.

Hence, their results radically differed from those reported for Neolithic and Bronze age domestic chicken exploitation by Xiang et al. (2014), Zhou (1981), and others. For the archaeological Phasianidae remains at Nanzhuangtou, Cishan, and Wangyin, Xiang et al. (2014) identified by ancient DNA analysis 11 bones belonging to chicken or *Gallus* junglefowl. In contrast, Eda et al. (2016) morphologically classified all 81 bones as nonchicken. These studies markedly differ in terms of the presence or absence of chicken and other Phasianidae bones. Xiang et al. (2014) analyzed at least two canid bones (Peters et al., 2015), more specifically the right canid metacarpi (Eda et al., 2016), and demonstrated them as “typical ancient chicken bones unearthed in northern China” (Xiang et al., 2014). Although Xiang et al. (2015a) insisted that they did not succeed in identifying the species of the canid bones and their identity has no bearing on the conclusions drawn by Xiang et al. (2014), Eda et al. (2016) stated that these bones should be considered “typical” and indicative of the reliability of the samples used by Xiang et al. (2014).

Eda et al. (2016) studied five tarsometatarsi from Cishan, which were probably identified by Zhou (1981) as domestic chicken. All bones had a medial plantar crest absent from the bones of chicken and red junglefowl and were identified as “nonchicken” bones (Eda et al., 2016). Zhou (1981) mentioned that the “oldest domestic chicken in the world” was from Cishan. However, he only stated that the specimens resembled wild red junglefowl in shape and did not explain the criteria for distinguishing chicken bones from those of indigenous birds from northern China. Moreover, Zhou (1981) displayed photographs of four tarsometatarsi with medial plantar crests (Plate 9.1–9.4) and designated them domestic chicken bones. However, no chickens or red junglefowl have a medial planter crest and these bones were obviously misidentified (Eda et al., 2016). The misidentification of tarsometatarsus is more critical than that of other bone elements because Zhou (1981) proposed chicken domestication at Cishan based on measurements and male-biased sex ratios in tarsometatarsus. Therefore, chicken domestication at Cishan is unsubstantiated (Eda et al., 2016). Although chicken bones have been discovered in at least 52 archaeological layers from 44 Neolithic sites and 18 layers from 12 Bronze Age sites in China, these records should be comprehensively reexamined (Eda et al., 2016).

### Early Holocene domestic chicken bones in Europe

Pleistocene and early Holocene *G. gallus* bones were also reported in Europe. Boev (1995) reviewed the Pleistocene and early Holocene archaeological *Gallus* bones from Moldova, Ukraine, Russia, Crimea, Georgia, Armenia, and Romania and proposed that a glacial refuge may have occurred in the southern Ukraine and Transcaucasus regions, allowing the domestication of the palaeolithic fowl there. In contrast, Mlíkovský (2002) stated that *Gallus* wild fowl could have been absent in Europe during the Würm III glaciation (ca. 70,000–10,000 years ago) and the middle Holocene. *Gallus gallus* and other bones dating from the late Pleistocene and early Holocene were found in France, England, Germany, Croatia, Ukraine, Romania, and Greece, with no reliable records in the assigned strata and/or species identification (Mlíkovský, 2002). Domestic chicken bones from the early and middle Holocene have also been reported at certain archaeological sites in Bulgaria. The oldest record was from Hotnista (ca. 5000 BC), in which identified bones were large (~3 kg) and originated from domesticated animals (Boev, 2009). However, Pitt et al. (2016) showed that modern and middle Holocene environmental conditions in Bulgaria were (and are) suboptimal for red junglefowl. Kyselý (2010) argued that these early findings were modest and incoherent and the unusually early dates reported for the European sites should be verified (Kyselý, 2010).

### Summary

Thus far, domestic chicken bones have been reported from the early Holocene, for example Nanzhuangtou (northern China, ca. 10,000 years ago; Xiang et al., 2014), Cishan (northern China, ca. 8,000 years ago; Zhou, 1981), and Hotnista (Bulgaria, ca. 7,000 years ago; Boev, 2009) and in several middle Holocene sites in northern China and Europe (reviewed in West and Zhou, 1988; Boev, 1995; Mlíkovský, 2002; Kyselý, 2010). However, the authenticity of these discoveries remains controversial (e.g., Kyselý, 2010; Eda et al., 2016; Peters et al., 2016).

## Future Perspectives for Studies of Origin of Domestic Chicken

### Reevaluation of the existence of domestic chicken at Mohenjo-Daro

The existence of domestic chicken in Indus Valley ca. 2000 B.C. was taken to be an established fact after Zeuner’s (1963) report. Nevertheless, none of the bones was identified as *G. g. domesticus* or *G. domesticus* in the original description of Mohenjo-Daro. Instead, they were designated “?*Gallus* sp.” (Sewell and Guha, 1931). The authors defined no criteria to distinguish domestic chicken bones from those of local indigenous Phasianidae species. Hence, it may be said that the identification of “domestic chicken” was unacceptable for the contemporaneous standard. Even if the bones were derived from domestic chickens, it should be verified that they originated from ca. 2000 BC. A femur from Mohenjo-Daro measured 103 mm (Sewell and Guha, 1931), which was larger than those of wild male red junglefowl (~0.7–1.5 kg; range, 74.72–80.04 mm; mean, 76.54 mm; *n* = 13) and captive male red junglefowl (range, 69.82–80.74 mm; mean, 73.91 mm; *n* = 24) (Eda, 2020) but similar to those of male Leghorn and Plymouth Rock (both ~3.4 kg).

It is difficult to regard the seals and figurines of chicken-like creatures as solid evidence for the existence of domestic chicken in the Harappan culture. Similarly, it is difficult to consider artistic representations of turtles, monkeys, and rhinoceros in Mohenjo-Daro as evidence of their domestication. Therefore, these discoveries merely suggest that the people during that period recognized similar creatures and the relationships among them. The environmental conditions of Mohenjo-Daro were speculated to be outside of the requirements of red junglefowl during the middle and late Holocene (Pitt et al., 2016). To confirm the existence of domestic chicken at certain early and middle Holocene archaeological sites, radiocarbon dating and accurate species identification of each bone sample are required.

### Phasianidae bone research in Southeast Asia

According to environmental considerations, red junglefowl domestication might have occurred within the native range of the species (Pitt et al., 2016). The latest molecular findings pointed to candidate sites in the distribution ranges of *G. g. spadiceus* in western Thailand, the Malaysian Peninsula, and eastern Myanmar, with the time of divergence between domestic chicken and *G. g. spadiceus* being estimated as 9,500 ± 3,300 years ago (Wang et al., 2020). The divergence estimate does not determine the origins of domestication but a split between the lineages leading to *spadiceus* and the ancestors of domestic chickens, which, at this point, were likely wild birds. The oldest chicken bones were thought to be intermixed with red junglefowl bones from the Holocene archaeological sites in Southeast Asia. However, reports of bird remains in Southeast Asia are scarce and prehistoric chicken and red junglefowl exploitation is obscure (Storey et al., 2012; Eda et al., 2019). Bone dating and species identification are essential to identify the oldest chickens in the world.

In regards to bone dating, the majority of the zooarchaeological specimens were dated using stratigraphic and/or contextual evidence. However, chicken bones can easily move between occupation phases; hence, precautions are required if samples were from sites with overlaying building structures or archaeological strata (Flink et al. 2014). For example, Flink et al. (2014) directly dated a chicken bone found from Iron Age La Tène C/D contexts (280–15 BC) and revealed the bone was actually from the early modern or modern period (1800 ± 30 AD). To confirm the age, direct radiocarbon dating of specimens are ideal, although it requires some destruction of the samples.

In regards to species identification, 43 Phasianidae fowl/pheasant species inhabit Southeast Asia. As far as I know, no morphological criteria have been established to distinguish chicken and red junglefowl from other indigenous fowl/pheasant bones. Ancient DNA analysis was used to effectively identify archaeological Phasianidae bones (Storey et al., 2012; Xiang et al., 2014; Prendergast et al., 2017; Barton et al., 2020). For the archaeological sites in Southeast Asia, Storey et al. (2012) analyzed the mitochondrial DNA control region of 10 “chicken” (including a stork coracoid; Eda et al., 2019) samples from Ban Non Wat (central Thailand, 3750–1500 BP) and produced two reliable and reproductible *G. gallus* sequences. The low success rate of the analysis could be due to the humid and warm temperature in Southeast Asia. Prendergast et al. (2017) analyzed morphologically identified chicken or Phasianidae bones from eight eastern African archaeological sites using ancient DNA analysis. They only succeeded to identify 6 (including five chicken and one hornbill) of 28 bones by polymerase chain reaction-based analysis. Then, they reanalyzed 19 of the specimens, which the previous analysis approach failed to identify, using high-throughput (shotgun) sequencing combined with BLAST-based computational analysis, and succeeded to identify six samples (including two *Gallus* and four indigenous pheasants) at the genus level (Prendergast et al., 2017). The high-throughput sequencing approach would be also useful for the Phasianidae bone identification in Southeast Asia. Moreover, Eda et al. (2020) found collagen peptide peaks, which were useful for discriminating domestic chicken and red junglefowl from indigenous Japanese pheasants, and successfully identified archaeological Phasianidae bones from a Japanese archaeological site. Some of the advantages of using bone collagen over DNA for analyzing archaeological samples include a higher success rate, need of a smaller amount of sample, and lower cost (Buckley et al., 2010). To date, certain collagen peptide peaks have been identified as being unique to domestic chickens and red junglefowls (Eda et al., 2020), which could also be useful to identify those zooarchaeological bones from Southeast Asia.

### Beyond the dichotomy of domestic chicken or wild red junglefowl

**Figure 6.**
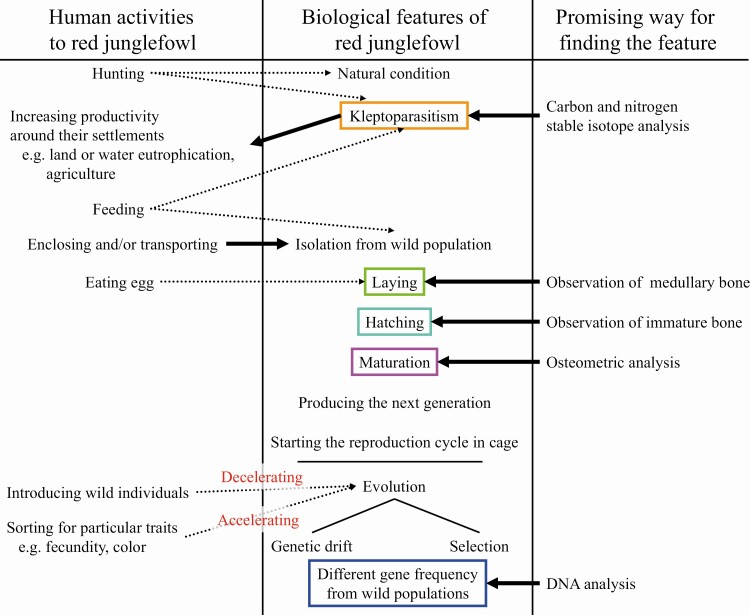
Plausible relationship history between humans and red junglefowl at various domestication stages.

It is a major challenge to determine if archaeological red junglefowl bones from Southeast Asia were of wild or domestic origin. For example, although Storey et al. (2012) found *G. gallus* sequences from Thai archaeological deposits dating approximately 2500 BP and 1550 BP, the data were insufficient to say that the bones were from domestic chicken, given there were no differences between mtDNA sequences of wild and domestic red junglefowl, in particular at the earliest stage of the domestication process (Figure 6). Red junglefowl excavated from various archaeological sites and different periods were not all necessarily at the same stage of domestication. Pure wild junglefowl used to be hunted. If people fed junglefowl, similar to what is suggested in northern China for common pheasants (Barton et al., 2020), the stable isotope ratios of nitrogen and carbon in red junglefowl bones could be different from wild individuals. This distinction might have occurred long before humans began breeding chickens as these animals were already using the resources near human settlements and crop fields.

Even if certain birds were isolated from the wild population via enclosure and/or long-distance transport, successful breeding was nonetheless required to maintain the population. In Phasianidae and most other birds, the medullary bone, a secondary bony structure, forms in medullary cavity in females at ~1 month before and after laying (Simkiss, 1961). Therefore, the appearance of the medullary bone suggests the presence of multiple mature individuals at that location during the breeding season. A high frequency of medullary bone suggests that the environment at the site was conducive to laying. Moreover, the breeding cycle could not start if humans consumed all the eggs laid by the chickens and it might have taken some time before an environment suitable to hatching was established. Chick mortality is in general high; thus, it would be expected for immature bird bones to be detected among the archaeological sites of a society that had prepared an appropriate environment for hatching. Overall, chicken breeding technology might have been introduced in areas where medullary bone and immature bones appeared simultaneously. In the Neolithic and Bronze Age sites of northern China, “candidate chicken” bones were found in sets with Phasianidae bones, including medullary bone and immature Phasianidae bones (Eda et al., 2016).

When the next generation of a breeding population was born, environmentally plastic phenotypic traits, such as bone size and proportions, may have differed from those of the wild population. Modern captive offspring of red junglefowl were generally smaller than wild red junglefowl, but the distal part of the tibiotarsus was thicker and parts of the wing bone were thinner in the former than in the latter (Eda, 2020). Similar morphological differences may have occurred between ancient wild and captive red junglefowl in the earliest stages of domestication (Eda, 2020). Geometric morphometrics would be useful to explore whether such morphological changes occurred in the early stage of domestication.

As domestication progresses further, artificial selection of individuals that lay more eggs, grow larger, and have a particular color of skin and feather could be envisioned. However, artificial selection would, at the same time, accelerate the rate of genetic drift by decreasing the effective number of individuals attending to breeding. Considering the genetic processes with the greatest potential impact on domestication, changes resulting from artificial selection are directional, whereas genetic drift produces random changes in gene frequencies (Price, 1984). Thus, selection and genetic drift may cause gene variants to disappear completely, thereby reducing genetic variation but also increasing the frequency of initially rare alleles. If a certain gene frequency within a domestic population is changed compared with the wild population, ancient DNA analysis can find whether the population could be domesticated in the original distribution area of the red junglefowl. In contrast, the introduction of wild individuals decelerates both adaptation to artificial environments and the rate of genetic drift.

Genetically determined traits are expected to differ from those of wild populations later in the domestication process. Flink et al. (2014) studied *BCDO2* and a putative domestication gene (thyroid-stimulating hormone receptor, *TSHR*), thought to be linked to photoperiod and reproduction mechanisms, from 80 European ancient chicken bones dated ~280 BC to the 18th century AD. They suggested that the commonality of yellow skin in Western breeds and the near fixation of a missense mutation within *TSHR* sequence in all modern chickens took place just past 500 years ago. With additional ancient DNA data and Bayesian statistical framework, Loog et al. (2017) showed that strong selection on variant *TSHR* allele began around 1,100 years ago, coincident with archaeological evidence for intensified chicken production and documented changes in egg and chicken consumption. Intriguingly, Wang et al. (2020) reported that the variant *TSHR* allele was found at high frequency in *G. g. spadiceus* (94.0%), whereas it had a frequency of only 5.4% in other red junglefowl subspecies. Further analysis of ancient genomes from chicken and red junglefowl spanning a wide timeframe and geographic areas are required to explain this pattern. Moreover, genome-wide studies of domestic chickens and red junglefowl have revealed some genes bearing selection associated with color phenotype, as well as the regulation of growth, metabolism, and reproduction, and the development of the nervous system, muscle, and bone (Huang et al. 2020, Wang et al. 2020). The selection on these traits also could be revealed by the extensive ancient DNA analysis of chicken bones using high-throughput sequencing technology.

## Conclusion

The origin of domestic chicken has been investigated mainly by modern biological and zooarchaeological approaches. The modern biological approach revealed that a red junglefowl subspecies is the main wild ancestor of the domestic chicken. However, other red junglefowl subspecies and wild junglefowl species also contributed to the modern domestic chicken genetic profile. In contrast, zooarchaeological methodology failed to detect archaeological bones that could be reliably identified as those derived from the “oldest domestic chicken”. Further zooarchaeological studies on the early and middle Holocene Phasianidae bones of Southeast Asia are required to make this determination. Analysis of archaeological red junglefowl bones from different perspectives should clarify the roles of these animals and their relationships with humans in each region and time period. Domestic chickens have recently been bred to provide meat and eggs worldwide (FAO, 2020). However, their principle ancestor, red junglefowl, weighs <1 kg and lays only four to eight eggs per year (Lawler, 2015). Elucidation of the origin of chicken domestication may provide useful insights into why red junglefowl rather than other bird taxa are the most common poultry and among the most commonly domesticated animals worldwide.

## About the Author

Masaki Eda studied archeology at the University of Tsukuba and ecology and molecular biology at the Graduate School of Agricultural and Life Sciences, University of Tokyo. After working as a JSPS Research Fellow at Kyushu University, he taught human anatomy at Tottori University as an assistant professor. He has worked at Hokkaido University Museum since 2012. Incorporating the viewpoints and approaches of each field, he mainly studies archaeological bird bones. He specializes in archaeozoology to elucidate the paleoecology of birds and zooarchaeology to reconstruct human lives with the knowledge they provide.
